# Machine Learning-Based Prediction of Short-Term Mortality After Coronary Artery Bypass Grafting: A Retrospective Cohort Study

**DOI:** 10.3390/biomedicines13082023

**Published:** 2025-08-19

**Authors:** Islam Salikhanov, Volker Roth, Brigitta Gahl, Gregory Reid, Rosa Kolb, Daniel Dimanski, Bettina Kowol, Brian M. Mawad, Oliver Reuthebuch, Denis Berdajs

**Affiliations:** 1 Department of Cardiac Surgery, University Hospital Basel, University of Basel, 4031 Basel, Switzerland; 2 Department of Mathematics and Informatics, University of Basel, 4056 Basel, Switzerland; 3 Surgical Outcome Research Center Basel, University Hospital Basel, University of Basel, 4031 Basel, Switzerland; 4 Department of Biomedical Engineering, University of Basel, 4123 Basel, Switzerland

**Keywords:** CABG, EuroSCORE II, machine learning, mortality prediction, postoperative variables, risk stratification

## Abstract

**Objectives:** This study aimed to develop and validate a machine learning (ML) algorithm to predict 30-day mortality following isolated coronary artery bypass grafting (CABG) and to compare its performance against the widely used European System for Cardiac Operative Risk Evaluation II (EuroSCORE II) risk prediction model. **Methods:** In this retrospective study, we included consecutive adult patients who underwent isolated CABG between January 2009 and December 2022. Three predictive models were compared: (1) EuroSCORE II variables alone (baseline), (2) EuroSCORE II combined with additional preoperative variables (Model I), and (3) EuroSCORE II plus preoperative and postoperative variables available within five days after surgery (Model II). Logistic Regression (LR), Random Forest (RF), and Neural Network (NN) were employed and validated. Predictive accuracy was assessed using the area under the receiver operating characteristic curve (AUC) and specificity at 85% sensitivity. **Results:** Among the 3483 patients included, the mean age was 66.2 years (SD 10.3), with an overall 30-day mortality rate of 2.5%. The mean EuroSCORE II was 3.12 (SD 4.8). Integrating additional preoperative variables significantly improved specificity at 85% sensitivity for both random forest (from 42% to 51%; *p* < 0.001) and NN (from 28% to 43%; *p* < 0.001) but not for LR. Incorporating preoperative along with postoperative data (Model II) further improved specificity to approximately 70% across all ML methods (*p* < 0.001). The most influential postoperative predictors included kidney failure, pulmonary complications, and myocardial infarction. **Conclusions:** ML models incorporating preoperative and postoperative variables significantly outperform the traditional EuroSCORE II in predicting short-term mortality following isolated CABG.

## 1. Introduction

Preoperative risk assessment is essential in cardiac surgery, guiding clinical decisions, resource allocation, and preparation for complications. In recent decades, various risk scoring systems have been introduced to support clinical decision-making, notably the EuroSCORE II, widely used across Europe [1]. Although broadly validated, a significant limitation of EuroSCORE II is its tendency to overestimate risk in certain high-risk patient subgroups, potentially leading to suboptimal therapeutic decisions and resource allocation [2]. Moreover, it has inherent limitations in capturing complex interactions between variables, such as the nuanced interplay between advanced age and impaired renal function, potentially leading to inaccuracies in predicting postoperative outcomes [2,3]. Machine learning (ML) offers a powerful alternative to traditional risk models by capturing complex, nonlinear relationships to improve predictive accuracy [4,5]. Despite progress in other fields, ML remains underutilized in cardiac surgery due to the need for large, high-quality datasets to develop reliable predictive models [6]. Larger datasets and improved computational resources have facilitated more complex and powerful predictive modeling in cardiology, showing substantial promise for clinical implementation [7,8,9,10,11]. Nonetheless, studies integrating comprehensive postoperative data remain scarce, despite their critical role in accurately predicting postoperative outcomes [12]. This represents a significant knowledge gap, particularly as EuroSCORE II approaches a decade since its implementation, necessitating re-evaluation and possible enhancement using contemporary analytical methods. In response to these challenges, the current study employs advanced ML techniques to develop and validate a comprehensive predictive model for short-term mortality in a large cohort of patients undergoing isolated coronary artery bypass grafting (CABG). We hypothesize that ML algorithms can predict 30-day mortality following isolated CABG at least as accurately as the established EuroSCORE II. The primary objective was to benchmark our ML algorithm in comparison to the well-established EuroSCORE II.

## 2. Materials and Methods

This retrospective study included adult patients (≥18 years) who underwent isolated CABG at the Department of Cardiac Surgery, University Hospital Basel, Switzerland, from January 2009 to December 2022. This study was approved by the Ethics Committee of Northwestern and Central Switzerland (EKNZ; project ID: 2020-02698, 5 January 2021), which waived the requirement for individual informed consent due to the retrospective nature of the analysis. Data were anonymized and securely stored in compliance with data protection regulations and hospital policies.

The primary objective was to assess the predictive performance of ML models in estimating 30-day mortality following isolated CABG. Patients were excluded from the study if they explicitly refused data usage or lacked essential preoperative or postoperative clinical data. Patient data were retrospectively extracted from comprehensive electronic medical records Dendrite Clinical Systems (Dendrite Clinical Systems Ltd., Henley-on-Thames, UK). A standardized data collection form was employed to ensure data consistency, including patient demographics, preoperative risk factors, and postoperative outcomes. Data quality control checks were regularly conducted by clinical and research personnel to ensure accuracy and consistency. Missing data were managed through imputation, utilizing the median for continuous variables and the most frequent category for categorical variables within the training set. Continuous variables were standardized (mean = 0, SD = 1), while categorical variables were numerically encoded for analytical purposes.

### Machine Learning

To evaluate and improve the prediction of 30-day mortality following isolated CABG, we developed and compared three distinct predictive models, each characterized by specific variable sets:

1. This model included standard variables included in the EuroSCORE II risk assessment (see Supplementary Table S1).
2. Model I (EuroSCORE II + additional preoperative variables): This extended model incorporated all the baseline EuroSCORE II variables with additional preoperative clinical parameters (see Supplementary Table S2).
3. Model II (EuroSCORE II + additional preoperative and postoperative variables): This comprehensive model incorporated all the variables from Model I, supplemented by detailed postoperative parameters and early postoperative complications recorded within the first five days following surgery (see Supplementary Table S3).

Three distinct ML approaches were developed and compared for mortality prediction:

- Logistic Regression (LR): A baseline statistical model using penalized maximum likelihood estimation.
- Random Forest (RF): An ensemble decision tree method implemented with default hyperparameters without further tuning.
- Neural Networks (NNs): A multi-layer perceptron architecture designed to identify complex nonlinear relationships.

A team of cardiac surgeons from the Department of Cardiac Surgery at the University Hospital Basel selected the additional preoperative and postoperative variables for inclusion in the ML models based on clinical judgment and their potential prognostic value in predicting 30-day mortality. To enable fair comparison across models, we used two performance measures, the area under the ROC curve (AUC) and the specificity at 85% sensitivity, both evaluated on a test set. The AUC serves as an integrative metric, capturing performance across all sensitivity–specificity thresholds, while the latter is better aligned with clinical priorities, namely minimizing false positives (“false alarms”) while ensuring that only a small proportion of critical cases (i.e., patients who died within 30 days) are missed. To mitigate class imbalance, we applied random undersampling of the majority class (survivors) in each training set. Model evaluation was conducted using repeated hold-out validation with 100 random 80/20 train–test splits with stratified outcome sampling. This approach was chosen over single train–test validation to provide more reliable and generalizable performance estimates, reducing the influence of specific data partitions on the results. Feature preprocessing included median imputation for continuous variables and mode imputation for categorical variables. Differences in model performance were assessed using paired Wilcoxon signed-rank tests.

The RF classifier was trained on 100 randomly chosen subsets, each with 68 patients per class (approximately 80% of the minority class size), ensuring balanced outcome representation. The remaining patients formed the test sets. Predictive performance was assessed using (i) AUC and (ii) specificity (1—false alarm rate) at the fixed sensitivity of 85% on the test splits. Paired Wilcoxon signed-rank tests evaluated model specificity difference, with significance defined as *p* < 0.05. Variable importance was evaluated using the Gini importance measure in RF models to determine the most predictive variables. All analyses were performed using R (version 4.1.2) and additional R-packages including “random Forest” (version 4.7-1.1), “sampling” (version 2.10), and “AUC” (version 0.3.2). The random forest package was used “off the shelf”, i.e., with the default parameter values. No further model selection was performed.

For comparison, a classical linear LR model with lasso (L1) regularization was trained on the same randomly selected subsets, using the R package gimlet (version 4.1-8). Unlike RF, LR models do not natively handle categorical variables; therefore, all factor variables were encoded using contrast coding. The regularization strength (sparsity penalty) was adaptively selected through an internal cross-validation loop within each training split.

Finally, an NN model in the form of a multi-layer perceptron with two hidden layers and ReLU activation functions was trained using the R package keras3 (version 1.3.0). As with the LR model, categorical variables were encoded using contrast coding. Tunable hyperparameters, including weight regularization constants, were optimized via an internal cross-validation loop within each training split.

## 3. Results

The mean age of the cohort was 66.2 ± 10.3 years, with a predominance of male patients (83%); the mean EuroSCORE II was 3.12 ± 4.8. The observed 30-day mortality for the entire cohort was 2.5% (*n* = 87).

### 3.1. Baseline Model: EuroSCORE II Variables

A detailed comparison of the clinical characteristics included in EuroSCORE II is provided in Table 1. Non-survivors were significantly older, had a lower preoperative ejection fraction and glomerular filtration rate, and showed a higher prevalence of preoperative risk factors including arteriopathy, reduced mobility, preoperative hemodynamic instability, diabetes, recent myocardial infarction, and higher operative urgency (all *p* < 0.001). The mean preoperative EuroSCORE II was markedly higher in the non-survivor group (13.8 ± 16.3 vs. 2.8 ± 4.7, *p* < 0.001).

### 3.2. Model I: EuroSCORE II Plus Preoperative Characteristics

A comparison of the additional preoperative variables included is presented in Table 2. Non-survivors exhibited higher rates of kidney disease (22% vs. 5.2%), unstable angina (39.1% vs. 22.1%), and cardiogenic shock (25.3% vs. 1.8%) (all *p* < 0.001). They also had higher preoperative creatinine levels (128.9 vs. 90.8 mg/dL; *p* < 0.001) and were more likely to require ventilator support before surgery (18.4% vs. 1.1%; *p* < 0.001).

### 3.3. Model II: EuroSCORE II Plus Pre- and Postoperative Characteristics

Finally, a comparison of the additional postoperative variables included in Model II is presented in Table 3. Compared to survivors, non-survivors had significantly higher postoperative peak creatinine levels (232.6 vs. 108.7 mg/dL; *p* < 0.001) and elevated cardiac biomarkers, including CK (3538 vs. 1062 mg/dL; *p* < 0.001), CK-MB (142.6 vs. 36.4 mg/dL; *p* < 0.001), and Troponin-T (4796 vs. 819 mg/dL; *p* < 0.001). Major complications such as postoperative myocardial infarction (21.8% vs. 1.9%, <0.001), stroke (16.1% vs. 1.9%, <0.001), kidney failure (46% vs. 5.7%, <0.001), and pulmonary complications (52.9% vs. 10.4%, <0.001) were also significantly more frequent among non-survivors.

### 3.4. Machine Learning: Model I vs. Baseline Model

For each ML method, differences in the predictive performance for these three feature subsets were analyzed separately using the AUC metric and the specificity at a sensitivity of 85% on the test splits. The latter calibration reflects a clinically meaningful threshold, prioritizing the minimization of false “alarms” (where alarm in this context refers to falsely assigning a survivor to the death group).

**Figure 1:**
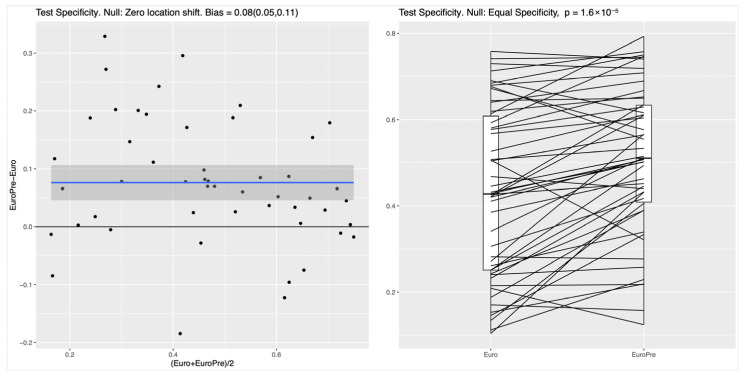
RF: Improvement in test specificity with additional preoperative features (Model I vs. baseline EuroSCORE II model). Legend: The figure illustrates the improvement in test specificity at 85% sensitivity when additional preoperative variables are added to the EuroSCORE II baseline model (*p* < 0.001). The left panel shows the paired differences in specificity for each of the 100 random train–test splits (EuroPre-Euro), with the blue line representing the mean difference (0.08) and its 95% confidence interval (0.05–0.11). The right panel displays paired boxplots comparing specificity values for the baseline model (“Euro”) versus the extended model (“EuroPre”), with each line connecting the results from the same data split.

**Figure 2:**
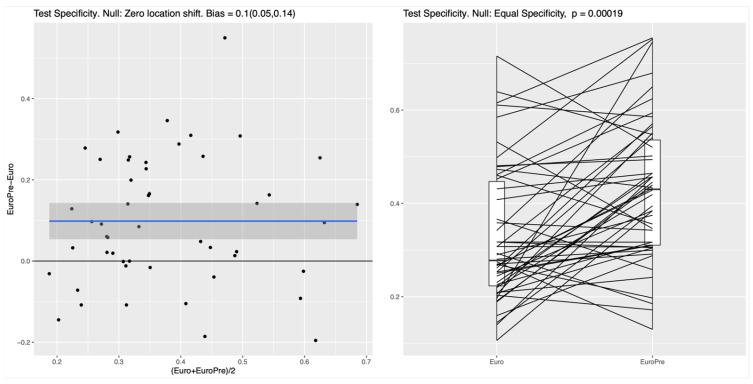
NN: Improvement in test specificity with additional preoperative features (Model I vs. baseline EuroSCORE II model). Legend: This figure shows the enhancement in test specificity at 85% sensitivity achieved by incorporating additional preoperative variables into the EuroSCORE II baseline model when using an NN classifier. In the majority of random splits, the classifiers trained on the features included in Model I demonstrated superior performance over those trained only on the EuroSCORE features. Specifically, for the RF, the median specificity increased from 42% (baseline model) to 51% for Model I, reflecting a consistent and clinically meaningful improvement in accurately identifying survivors. This improvement was statistically significant (*p* < 0.001). Similarly for the NN, the median specificity increased from 28% to 43% (*p* < 0.001). The blue line represents the fitted mean difference in test specificity between the two compared models across the range of average specificity, with the shaded gray area indicating the 95% confidence interval; the black horizontal line denotes no difference.

**Figure 3:**
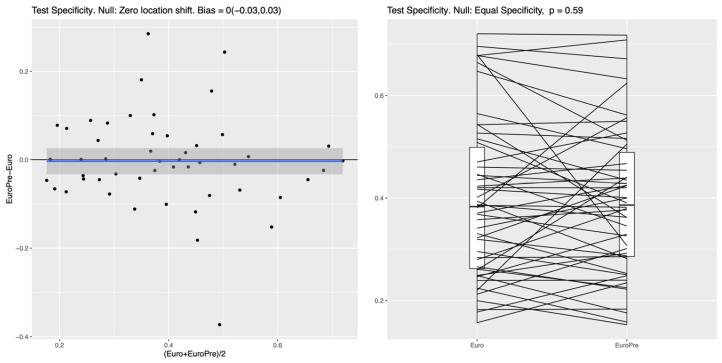
LR: No improvement in test specificity with additional preoperative features (LR, Model I vs. baseline EuroSCORE II model). Legend: This figure compares the test specificity of an LR model using only EuroSCORE II variables (“Euro”) with a model that includes additional preoperative features (“EuroPre”). Contrary to the nonlinear RF and NN classifiers, for the linear LR model, no specificity differences are observable. For the RF, variable importance was analyzed using the Gini index, revealing that among the additional preoperative features that are not included in the EuroSCORE variables, the most predictive feature in the extended set in Model I was cardiogenic shock, followed by “previousmi”/“previousmitime”, history of previous ventilation, and “crea”. The blue line represents the fitted mean difference in test specificity between the two compared models across the range of average specificity, with the shaded gray area indicating the 95% confidence interval; the black horizontal line denotes no difference.

For both the RF and the NN classifier, the specificity on the test set, measuring the ability to correctly identify survivors, was significantly higher for Model I compared to the EuroSCORE variables alone in the baseline model (*p*-values of roughly 10^−4^); see Figure 1 for the RF and Figure 2 for the NN. In the left panel of both figures, the blue line shows the mean difference in specificity, averaged over all randomly chosen training–test splits, which is approximately 0.08 (95%CI: 0.05–0.11) for the RF and 0.1 (95%CI 0.05–0.14) for the NN, indicating that the additional preoperative features in Model I indeed lead to a significantly increased specificity. For the linear LR model, no clear difference between the two feature subsets was detectable (see Figure 3), which suggests that the inherent nonlinearity of both the RF and the NN is essential for exploiting the full potential of the additional preoperative variables. The model comparison using the AUC metric shows essentially the same trends for all experiments, as shown in Figure S1, Figure S2 and Figure S3 in the Supplementary Materials.

### 3.5. Machine Learning: Model II vs. Baseline Model

**Figure 4:**
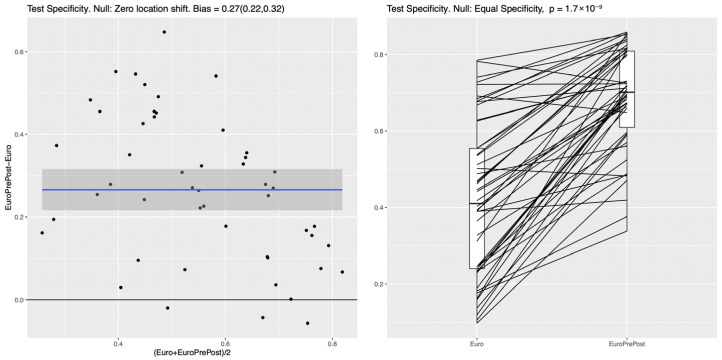
RF: Improvement in test specificity (RF, Model II vs. baseline EuroSCORE II model). Legend: This figure demonstrates the impact of incorporating both preoperative and postoperative variables (“EuroPrePost,” corresponding to Model II) compared to EuroSCORE II variables alone (“Euro”) on test specificity at 85% sensitivity using an RF classifier. Additional pre- and postoperative features (labeled “EuroPrePost” in the plot, corresponding to Model II in the text) vs. EuroSCORE variables alone (labeled “Euro” in the plot corresponding to the baseline model). The left panel shows a clear average specificity difference between the two feature sets of 0.27 (95%CI: 0.22, 0.32). The right panel indicates that in almost any random data split, the full feature set leads to a substantially increased performance. The median specificity at 85% sensitivity increased from 0.41 to 0.7 (*p* < 0.001). The blue line represents the fitted mean difference in test specificity between the two compared models across the range of average specificity, with the shaded gray area indicating the 95% confidence interval; the black horizontal line denotes no difference.

After including additional postoperative features, all ML classifiers demonstrated much better specificity compared to the baseline model. The mean difference for the RF is 0.27 (95%CI: 0.22–0.32), which is similar to the obtained differences for the NN (0.26) (95%CI: 0.22–0.31) and the LR (0.26) (95%CI: 0.19–0.32). This result indicates that in this experiment, all ML methods (i.e., LR, RF, and NN) behave very similarly, and that they all have clearly higher specificity for the full set of pre- and postoperative features considered, as shown in Figure 4. Similar improvements in test specificity with the inclusion of postoperative features were observed for the NN and LR models, as shown in Figures S4 and S5 in the Supplementary Materials.

## 4. Discussion

In our study, we demonstrated that incorporating postoperative cardiovascular variables into machine learning models significantly improves the prediction of early adverse events. Accurate preoperative risk assessment remains essential in cardiac surgery to guide clinical decision-making and optimize patient outcomes. Traditional models like EuroSCORE II, while widely utilized, have limitations in terms of predictive accuracy, particularly due to their reliance on linear assumptions and a predefined set of variables [1,13,14]. Moreover, although EuroSCORE II demonstrates good discriminative ability, it has been shown to significantly overestimate mortality risk, particularly among younger patients [15].

In both nonlinear ML methods investigated, the RF and the NN, the integration of preoperative variables with the baseline EuroSCORE II variables (Model I) resulted in a notable improvement in specificity for predicting mortality [16]. In the RF model, specificity improved from 42% to 51% while maintaining a fixed sensitivity of 85%, while comparable improvements were observed for the NN. In contrast, no significant performance gain was seen with the linear LR model, suggesting that it lacks the capacity to capture the complex relationships embedded in the expanded feature set. Between the two nonlinear classifiers (i.e., the RF and the NN), we could not identify any noticeable performance differences in our experimental setting. However, the RF turned out to be simpler to implement, required no parameter tuning, and offered transparent feature importance for easy interpretation.

Further augmentation with the implementation of postoperative variables (Model II) augmented the specificity of the prediction of mortality to 70%, underscoring the substantial value of the postoperative data in mortality prediction. This approach allows for dynamic risk reassessment, reflecting how complications or physiological responses during and immediately after surgery influence short-term mortality risk. By integrating postoperative data, such as renal function, biomarkers, and early complications, Model II enables clinicians to update prognostic assessments in real time, offering a more nuanced understanding of each patient’s risk profile. The substantial improvement in specificity achieved by our ML models may translate into a clinically meaningful reduction in false positive classifications. By more accurately identifying lower-risk patients, the ML model could help avoid unnecessary interventions, reduce patient burden, and improve resource allocation, all while maintaining high sensitivity. Additionally, variable importance analysis within the RF model identified postoperative kidney failure as the strongest predictor of 30-day mortality, followed by pulmonary complications and postoperative myocardial infarction, underscoring the prognostic relevance of early postoperative events in short-term outcome prediction. To facilitate clinical translation, the developed ML models could be implemented as an online risk calculator or integrated into electronic health records, providing clinicians with real-time, data-driven mortality risk estimates to support preoperative decision-making and patient counseling.

Our findings align with and expand upon previous studies on ML in cardiac surgery risk prediction, further strengthening the evidence for its integration. Recent research supports our findings that ML models offer superior predictive performance compared to traditional LR-based scores. Benedetto et al. demonstrated in a meta-analysis that ML algorithms consistently showed better discrimination in predicting postoperative mortality after cardiac surgery [5]. Similarly, Weiss et al. showed that institution-specific ML models trained on multimodal electronic health records significantly outperformed standard STS risk scores in individual mortality prediction across various cardiac procedures [17]. By incorporating both preoperative and postoperative variables, our model enables a more comprehensive and dynamic risk assessment, an approach particularly relevant in light of the ongoing development of EuroSCORE 3, which is expected to integrate ML [5]. Overall, multiple previous studies reported superior performance of various ML models over EuroSCORE II in predicting postoperative mortality after cardiac surgery [18,19,20,21]. A recent systematic review and meta-analysis demonstrated that machine learning models significantly outperform traditional logistic regression in predicting operative mortality after cardiac surgery, although the clinical impact of this improvement remains uncertain [5].

The inclusion of over 3000 consecutive patients over a 13-year period provides substantial statistical power and real-world relevance. Furthermore, the stepwise integration of preoperative and postoperative variables offers valuable insights into their respective contributions to the predictive performance of machine learning models. However, this study has several limitations. The retrospective design of this study may introduce inherent biases related to data collection and patient selection. Secondly, while our models demonstrated improved specificity, the generalizability of these findings to other institutions or patient populations remains to be established. Third, the lack of external validation using an independent dataset limits the generalizability of our findings and should be addressed in future multicenter studies. Prospective multicenter studies are needed to validate and refine these ML models, explore real-time data integration for dynamic risk prediction, and assess practical implementation, including system integration, staff training, and cost-effectiveness. Moreover, the use of default hyperparameters for the random forest model and limited tuning for the neural network may have constrained the full predictive potential of these models. We did not perform systematic ablation analysis to quantify the incremental value of individual variable groups, which would provide more precise guidance for clinical implementation. Additionally, while our machine learning models demonstrated improved predictive performance, the lack of explainable AI approaches limits their clinical adoptability. Finally, due to a significant class imbalance (low mortality rate of ~2.5%), we prioritized clinically relevant metrics (AUC and specificity at fixed sensitivity) and thus did not report the accuracy or F1-score, which may have limited interpretability in this context.

## 5. Conclusions

Incorporating both preoperative and postoperative variables into ML models significantly enhances the specificity of 30-day mortality predictions following CABG compared to the EuroSCORE II. These findings underscore the potential of ML to augment traditional risk assessment tools in cardiac surgery, highlighting the need for further research to validate and implement these models in diverse clinical settings.

## Figures and Tables

**Table 1:** EuroSCORE II variables included in the model.

Variable	Alive (*n* = 3396)	Dead (*n* = 87)	*p*-Value
Males	2841 (83.7%)	65 (74.7%)	0.02
Females	555 (16.3%)	22 (25.3%)	0.02
Age	66.09 (9.84)	71.44 (9.80)	<0.001
GFR	88.35 (33.63)	69.16 (37.26)	<0.001
EF preop	52.8 (11.38)	43.7 (15.20)	<0.001
MPAP	0.10 (0.43)	0.28 (0.62)	<0.001
COPD (Grade 3–4)	130 (3.9%)	6 (6.9%)	<0.001
Arteriopathy	712 (21.0%)	39 (44.8%)	<0.001
Mobility limitation	110 (3.2%)	13 (14.9%)	<0.001
Previous operations	78 (1.8%)	2 (2.3%)	0.98
Preop instability	238 (7.0%)	31 (35.6%)	<0.001
Diabetes	1270 (37.3%)	43 (49.4%)	<0.001
CCS (Grade 3–4)	1620 (47.7%)	57 (65.5%)	<0.001
Recent MI	1479 (43.6%)	57 (65.5%)	<0.001
NYHA (Grade 3–4)	662 (19.4%)	41 (47.1%)	<0.001
Urgency	961 (28.2%)	50 (57.4%)	<0.001
EuroSCORE	2.84 (4.72)	13.76 (16.28)	<0.001

Legend: The table presents the distribution of standard EuroSCORE II variables among survivors and non-survivors after isolated CABG. As none of the patients had endocarditis or thoracic surgery and all the patients had isolated CABG, these variables were excluded. Values are reported as mean (SD) for continuous variables and *n* (%) for categorical variables. Abbreviations: GFR—glomerular filtration rate, EF—ejection fraction, MPAP—mean pulmonary artery pressure, COPD—chronic obstructive pulmonary disease, CCS—Canadian Cardiovascular Society (Angina Classification), MI—myocardial infarction, NYHA—New York Heart Association (Heart Failure Classification).

**Table 2:** Preoperative variables included in the model.

Variable	Alive (*n* = 3396)	Dead (*n* = 87)	*p*-Value
Height (cm)	172.22 (8.37)	168.37 (9.70)	<0.001
Weight (kg)	82.08 (15.05)	79.57 (18.91)	0.12
BMI	27.64 (5.19)	27.91 (5.58)	0.63
Body surface area (m^2^)	1.95 (0.19)	1.89 (0.24)	<0.001
Dyslipidemia	2615 (77%)	63 (72.4%)	<0.001
Hypertension	2932 (86.3%)	70 (80.5%)	<0.001
Atrial fibrillation	139 (4.1%)	3 (3.45%)	<0.001
TIA	101 (3%)	7 (8%)	<0.001
Family history	1519 (44.7%)	37 (42.5%)	<0.001
Smoker	847 (25%)	22 (25.3%)	0.12
Anti-coagulation drugs	3018 (88.9%)	78 (89.7%)	<0.001
Cancer	300 (8.8%)	11 (12.6%)	<0.001
PAD (none)	2916 (85.9%)	57 (65.5%)	<0.001
Kidney disease	178 (5.2%)	19 (22%)	<0.001
Last preoperative creatinine (mg/dL)	90.8 (51.9)	128.9 (129)	<0.001
Carotid stenosis	234 (6.9%)	10 (11.5%)	<0.001
Previous vascular surgery/amputation	187 (5.5%)	12 (13.8%)	<0.001
Previous MI	1525 (45%)	45 (51.7%)	<0.001
Ventilated preop	38 (1.1%)	16 (18.4%)	<0.001
Left or right heart catheterization	1119 (33%)	51 (58.2%)	<0.001
Perioperative PCI	87 (2.5%)	3 (3.5%)	0.14
Triple vessel disease	2187 (64.4%)	49 (56.3%)	0.38
Instable angina pectoris	751 (22.1%)	34 (39.1%)	<0.001
Cardiogenic shock	61 (1.8%)	22 (25.3%)	<0.001
MI <6 h before CABG	186 (5.5%)	21 (24.1%)	<0.001

Legend: The table summarizes the clinical and laboratory parameters collected preoperatively but not included in the original EuroSCORE II. Values are reported as mean (SD) for continuous variables and *n* (%) for categorical variables. Abbreviations: MI—myocardial infarction; PAD—peripheral artery disease; TIA—transient ischemic attack; PCI—percutaneous coronary intervention; CABG—coronary artery bypass grafting.

**Table 3:** Postoperative variables included in the model.

Variable	Alive (*n* = 3396)	Dead (*n* = 87)	*p*-Value
Max. creatinine value (*n*)	108.7 (75.22)	232.6 (159.00)	<0.001
Max. CK value (U/L)	1062 (1919.42)	3538 (4646.85)	<0.001
Max. CK-MB value (U/L)	36.4 (67.93)	142.6 (185.11)	<0.001
Max. Troponin-T (ng/mL)	819 (1969.10)	4796 (6571.41)	<0.001
Perioperative MI	65 (1.91%)	19 (21.8%)	<0.001
Cardiac complications (none)	2509 (73.9%)	21 (24.1%)	<0.001
Stroke	64 (1.9%)	14 (16.1%)	<0.001
Neurological non-cerebral complications (none)	2848 (83.9%)	57 (65.5%)	<0.001
Kidney failure	193 (5.7%)	40 (46%)	<0.001
Pulmonary complication (none)	3043 (89.61%)	41 (47.1%)	<0.001
Other complications (none)	2905 (85.5%)	17 (19.5%)	<0.001

Legend: The table lists perioperative clinical and laboratory variables available within the first five days following surgery. Values are reported as mean (SD) for continuous variables and *n* (%) for categorical variables. This table presents postoperative variables compared between survivors and non-survivors following isolated CABG. Continuous variables are expressed as the mean (standard deviation), and categorical variables as a number (percentage). Abbreviations: CK—creatine kinase; CK-MB—creatine kinase–myocardial band; MI—myocardial infarction; ng/mL—nanograms per milliliter; U/L—units per liter.

## Data Availability

The original contributions presented in this study are included in the article/Supplementary Materials. Further inquiries can be directed to the corresponding author.

## Supplementary Materials

The following supporting information can be downloaded at: https://www.mdpi.com/article/10.3390/biomedicines13082023/s1, Figure S1. Random Forest: Comparison of AUC Between Model I (EuroScore II + additional Preoperative variables) and Baseline Model (EuroScore II); Figure S2. Neural Network: Comparison of AUC Between Model I (EuroScore II + additional Preoperative variables) and Baseline Model (EuroScore II); Figure S3. Logistic Regression: Comparison of AUC Between Model I (EuroScore variables + additional preoperative variables) and Baseline Model (EuroScore varaibles alone); Figure S4. Neural Network: Comparison of test specificity at 85% sensitivity between Model II (EuroScore variables + additional pre- and postoperative variables) and Baseline Model (EuroScore variables alone); Figure S5. Logistic Regression: Comparison of test specificity at 85% sensitivity between Model II (EuroScore variables + additional pre- and postoperative variables) and Baseline Model (EuroScore variables alone). Table S1. Euroscore II variables included in the model; Table S2. Preoperative variables included in the model; Table S3. Postoperative variables included in the model; Table S4: Comparison of 30-Day Mortality ML Models vs. State-of-the-Art Approaches.

## Abbreviations

The following abbreviations are used in this manuscript:

AF	Atrial Fibrillation
AP	Angina Pectoris
BSA	Body Surface Area
CABG	Coronary Artery Bypass Grafting
CAD	Coronary Artery Disease
CCS	Canadian Cardiovascular Society
CK-MB	Creatine Kinase–Myocardial Band
COPD	Chronic Obstructive Pulmonary Disease
CVI	Cerebrovascular Incident
DVT	Deep Vein Thrombosis
ECC	Extracorporeal Circulation
EF	Ejection Fraction
GFR	Glomerular Filtration Rate
ICD	Implantable Cardioverter Defibrillator
LR	Logistic Regression
MI	Myocardial Infarction
ML	Machine Learning
MOF	Multi-Organ Failure
MPAP	Mean Pulmonary Arterial Pressure
NB	Naive Bayes
NN	Neural Network
NP	Negative Predictive Value
NYHA	New York Heart Association
PAD	Peripheral Arterial Disease
PCI	Percutaneous Coronary Intervention
PPV	Positive Predictive Value
RF	Random Forest
SIRS	Systemic Inflammatory Response Syndrome
STS	Society of Thoracic Surgeons
UTI	Urinary Tract Infection
