# Supplementary material for: Machine Learning-Based Prediction of Short-Term Mortality After Coronary Artery Bypass Grafting: A Retrospective Cohort Study
Source: Biomedicines. 2025 Aug 19;13(8):2023. doi: 10.3390/biomedicines13082023 (PMC12383604; doi:10.3390/biomedicines13082023)
Supplement: Supplementary file 1 [file biomedicines-13-02023-s001.zip › biomedicines-3800483-supplementary.pdf]

Supplementary materials

Table S1. Euroscore II variables included in the model.

	**Variable**
**1**	Sex (Males)
**2**	Sex (Females)
**3**	Age
**4**	GFR
**5**	EF preop
**6**	MPAP
**7**	COPD (Grade 3-4)
**8**	Arteriopathy
**9**	Mobility limitation
**10**	Previous operations
**11**	Preop instability
**12**	Diabetes
**13**	CCS (Grade 3-4)
**14**	Recent MI
**15**	NYHA (Grade 3-4)
**16**	Urgency
**17**	EF
**18**	Euroscore

Legend: The table lists the standard EuroSCORE II variables among survivors and non-survivors after isolated CABG. As neither of patients had endocarditis, thoracic surgery, and all patients had isolated CABG, these variables were excluded. **Abbreviations: GFR** – Glomerular Filtration Rate, **EF** – Ejection Fraction, **MPAP** – Mean Pulmonary Artery Pressure, **BSA** – Body Surface Area, **COPD** – Chronic Obstructive Pulmonary Disease, **CCS** – Canadian Cardiovascular Society (Angina Classification), **MI** – Myocardial Infarction, **NYHA** – New York Heart Association (Heart Failure Classification), **CABG** – Coronary Artery Bypass Grafting.

Table S2. Preoperative variables included in the model.

	**Variable**
**1**	Height (cm)
**2**	Weight (kg)
**3**	BMI
**4**	Body Surface Area (m^2)
**5**	Dyslipidemia
**6**	Hypertension
**7**	Atrial fibrillation
**8**	TIA
**9**	Family history
**10**	Smoker
**11**	Anti-coagulation drugs
**12**	Cancer
**13**	PAD (none)
**14**	Kidney disease
**15**	Last pre-operative creatinine (mg/dl)
**16**	Carotid stenosis
**17**	Previous vascular surgery/amputation
**18**	Previous MI
**19**	Ventilated preop
**20**	Left- or right-heart catheterisation
**21**	Perioperative PCI
**22**	Triple vessel disease
**23**	Instable Angina-pectoris
**24**	Cardiogenic shock
**25**	MI <6 hours before CABG

Legend: The table lists clinical and laboratory parameters collected preoperatively but not included in the original EuroSCORE II. Abbreviations: **COPD** – Chronic Obstructive Pulmonary Disease; **CCS** – Canadian Cardiovascular Society; **MI** – Myocardial Infarction; **NYHA** – New York Heart Association; **BSA** – Body Surface Area; **PAD** – Peripheral Artery Disease; **TIA** – Transient Ischemic Attack; **PCI** – Percutaneous Coronary Intervention; **CABG** – Coronary Artery Bypass Grafting.

Table S3. Postoperative variables included in the model.

	Variable
**1**	Max. creatinin-value (n)
**2**	Max. CK-value (U/l)
**3**	Max. CK-MB value (U/l)
**4**	Max. Troponin-T (ng/ml)
**5**	Perioperative MI
**6**	Cardiac complications (none)
**7**	Stroke
**8**	Neurological non-cerebro complications (none)
**9**	Kidney failure
**10**	Pulmonary complication (none)
**11**	Other complications (none)

Legend: The table lists postoperative clinical and laboratory variables available within the first five days following surgery. Abbreviations: **CK** – Creatine Kinase; **CK-MB** – Creatine Kinase Myocardial Band; **MI** – Myocardial Infarction; **ng/ml** – Nanograms per millilitre; **U/l** – Units per litre; **CABG** – Coronary Artery Bypass Grafting.

Table S4. Comparison of 30-Day Mortality ML Models vs. State-of-the-Art Approaches.

Study (Year, Country)	Population (N, Setting)	ML Methods	Performance (AUC, Accuracy, Sensitivity, Specificity, etc.)
Salikhanov et al. (2025, Switzerland) – *This study*	Isolated CABG, 30-day mortality (N=3,483); single-center (Basel, CH)	EuroSCORE II (logistic) vs. ML models: Random Forest (RF), Neural Network (NN), Logistic Regression (LR); compared using EuroSCORE II variables, +additional pre-op, +post-op data	Adding pre-op features improved RF specificity from ~42% to 51% and NN from 28% to 43% at 85% sensitivity (no gain for LR). With post-op data included, all ML models achieved ~70% specificity at 85% sensitivity, significantly outperforming the EuroSCORE II baseline model in discrimination of 30-day mortality. (AUC increased by ~0.03–0.04 for RF/NN with added features, though baseline and final AUC not explicitly reported.)
Allyn et al. (2017, France)	Elective adult cardiac surgery (CABG, valve; N=6,520); single-center (Paris, FR)	Gradient Boosting, RF, SVM, Naïve Bayes; ensemble ("ML model") vs. EuroSCORE II and LR model	ML ensemble achieved AUC 0.795 for in-hospital mortality, outperforming EuroSCORE II (AUC 0.737) and a logistic regression model (AUC 0.742)[18]. Decision curve analysis indicated greater net clinical benefit for the ML model across relevant risk thresholds[18].
Molina et al. (2022, Colombia)	Adult cardiac surgery (mixed procedures; 2008–2018); single-center (Bogotá, CO)	Compared various ML models; best: Gradient Boosting (XGBoost) vs. EuroSCORE II	Best ML model (gradient boosting) AUC 0.755, vs. EuroSCORE II AUC 0.716[5]. Precision–recall (PR) AUC was 0.292 vs. 0.179 for EuroSCORE II[21]. The ML model showed higher discrimination and precision, but the AUC improvement was not statistically significant (p=0.318)[21].
Castela Forte et al. (2022, Netherlands)	Cardiac surgery (first-time CABG and/or valve; N=9,415); single center (Groningen, NL)	Multiple ML models using preoperative, intraoperative, and postoperative variables (ensemble approach)	Comprehensive perioperative ML models predicting 30-day mortality achieved AUROC ≈0.82 (95% CI ~0.78–0.86)[12]. Notably, using all pre/intra/postoperative data yielded AUROC ~0.82 for 30-day mortality, similar to models using only postoperative data (AUROC ~0.78)[12]. Postoperative factors (e.g. markers of metabolic and renal dysfunction) were among the top contributors to risk[12].
Benedetto et al. (2022, Systematic Review)	Meta-analysis of 15 studies (2012–2020) in cardiac surgery populations (mixed CABG/valve)	Various ML techniques vs. logistic EuroSCORE/STS models (best ML model from each study)	Pooled results showed ML models had significantly better discrimination than logistic regression: C-statistic ~0.88 for ML vs. ~0.81 for LR[5]. In meta-analysis, the best ML algorithms outperformed traditional risk scores on average (ΔAUC +0.07)[5], though authors note the clinical impact of this improvement is modest[5].
Sinha et al. (2023, UK)	Adult cardiac surgery (all types; N=227,087); multi-center national database (UK, 2012–2019)	EuroSCORE II vs. retrained Logistic Regression vs. ML models: RF, Neural Network, XGBoost, weighted SVM (using 18	In an external 30% test set, XGBoost achieved AUC ~0.834 and RF ~0.833, compared to EuroSCORE II's AUC ~0.818[19]. The ML models slightly improved F1-score (e.g. XGBoost F1 ~0.28 vs. 0.24 for EuroSCORE II)[19]. Calibration

		EuroSCORE II variables for all models)	was similar, but EuroSCORE II exhibited risk overestimation; ML models (especially XGBoost/RF) showed less calibration drift and a greater net benefit in decision curve analysis[19].
Weiss et al. (2023, USA)	Adult cardiac surgery (CABG, valve, etc.; N=6,392); single-center (New York, USA)	Extreme Gradient Boosting (XGBoost) model using rich institutional EHR data ("multi-modal" features) vs. STS risk scores	The institution-specific XGBoost model had AUC 0.978 for in-hospital mortality[17]. At the optimal threshold, overall accuracy was ~98.6%, with precision ~75.6% and recall ~79.5%[17]. This ML model substantially outperformed the standard STS risk score for mortality across all surgery subtypes in the cohort[17], highlighting the value of tailored, data-rich models for individual centers.
Xu et al. (2024, China)	Isolated CABG surgery, in-hospital mortality; ~4,764 training + external validation (N=1,733 & 415); multi-center (3 hospitals)	Ensemble "XCL" model (bagging of XGBoost, CatBoost, LightGBM) vs. EuroSCORE II	The ensemble ML model achieved AUC 0.9145 on internal validation[20], with high sensitivity (~94%) and moderate specificity (~71%) at the chosen operating point[20]. It remained the top performer in two external test centers (AUC ~0.88–0.91)[20], demonstrating superior discrimination and net reclassification improvement over EuroSCORE II in predicting CABG mortality [20].

Legend: The table summarizes studies published within the last 10 years on machine learning models for predicting 30-day or in-hospital mortality after cardiac surgery, including CABG. It benchmarks our model against recent state-of-the-art approaches in terms of dataset, ML methods used, and predictive performance. **CABG –** Coronary artery bypass grafting; **EuroSCORE II** – European System for Cardiac Operative Risk Evaluation II; **STS** – Society of Thoracic Surgeons risk score; **ML** – machine learning; **RF** – random forest; **SVM** – support vector machine; **AUC** – area under **ROC** curve; **AUROC** – AUC; **PR AUC** – area under precision–recall curve; **CI** – confidence interval; **EHR** – electronic health records.

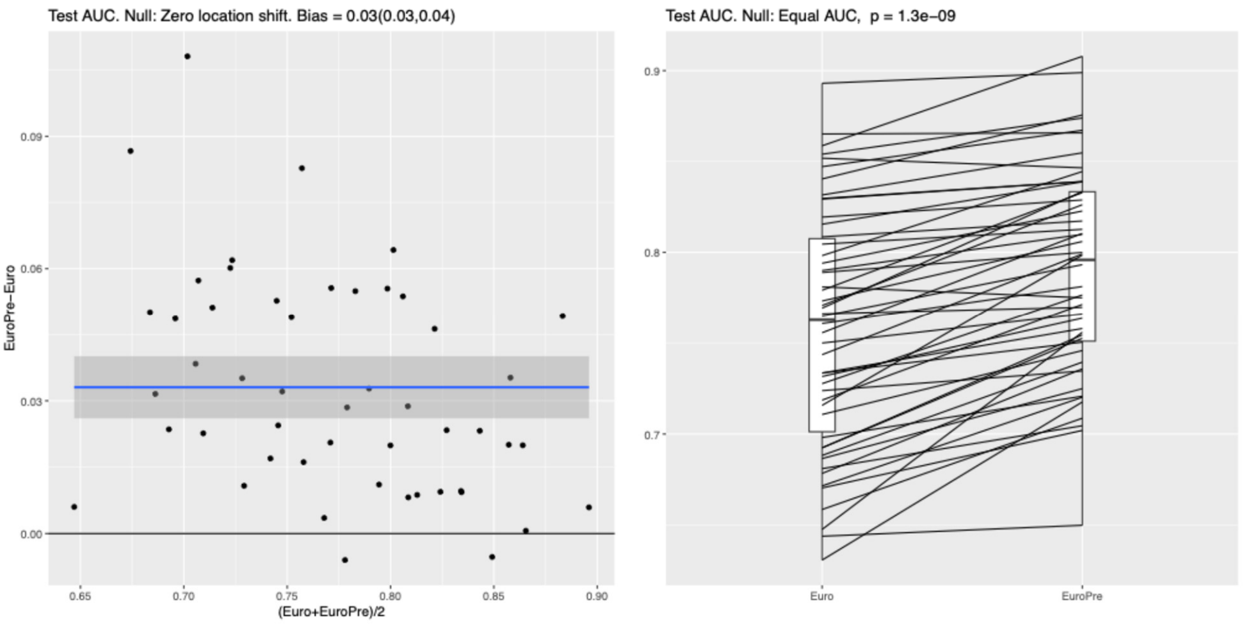

Figure S1. Random Forest: Comparison of AUC Between Model I (EuroScore II + additional Preoperative variables) and Baseline Model (EuroScore II). Legend: This figure compares AUC for the Random Forest classifier trained on the baseline EuroSCORE II variables ("Euro") and an extended feature set including additional preoperative variables ("EuroPre," Model I). The left panel displays the distribution of AUC differences across 100 random train-test splits, with an average improvement of 0.03 (95% CI: 0.03–0.04). The right panel shows paired AUC values for each data split, with lines connecting matched comparisons. The observed improvement is statistically significant ($p = 1.3 \times 10^{-9}$, Wilcoxon signed-rank test), demonstrating a consistent enhancement in discriminative performance when preoperative features are added to the baseline model.

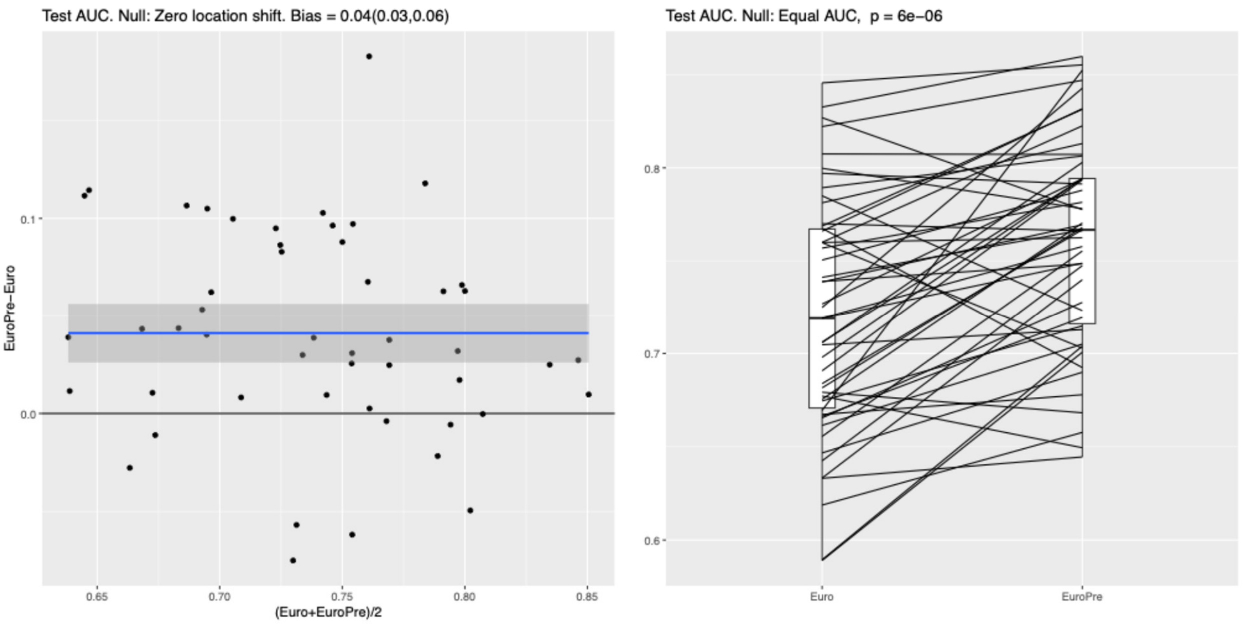

Figure S2. Neural Network: Comparison of AUC Between Model I (EuroScore II + additional Preoperative variables) and Baseline Model (EuroScore II). Legend: This figure compares AUC for a neural network classifier trained on the baseline EuroSCORE II variables ("Euro") versus an extended set including additional preoperative variables ("EuroPre," Model I). The left panel displays the paired AUC differences across 100 random train-test splits, with a mean improvement of 0.04 (95% CI: 0.03–0.06, blue line). The right panel presents matched AUC values with each line connecting results from the same data split. The improvement in AUC is statistically significant ($p = 6 \times 10^{-6}$, Wilcoxon signed-rank test), indicating that the inclusion of supplementary preoperative data enhances the model's discriminative ability.

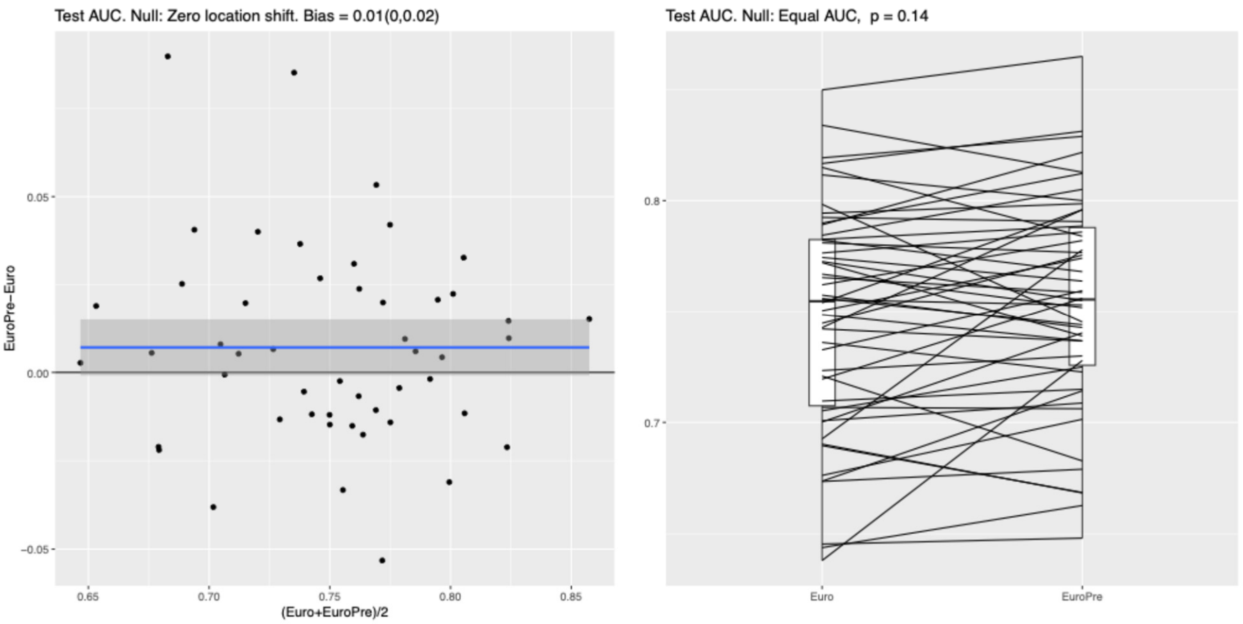

Figure S3. Logistic Regression: Comparison of AUC Between Model I (EuroScore variables + additional preoperative variables) and Baseline Model (EuroScore varaibles alone). Legend: This figure illustrates the comparison of AUC values between logistic regression models trained on EuroSCORE II variables alone ("Euro") and those incorporating additional preoperative features ("EuroPre," Model I). The left panel displays the paired AUC differences across 100 train-test splits, showing a mean improvement of 0.01 with a 95% confidence interval of (0.00–0.02). The right panel presents the AUC values for each model as boxplots with lines connecting paired results. No statistically significant improvement in AUC was observed with the extended feature set ($p = 0.14$, Wilcoxon signed-rank test), indicating that logistic regression fails to leverage the additional preoperative variables effectively compared to nonlinear classifiers.

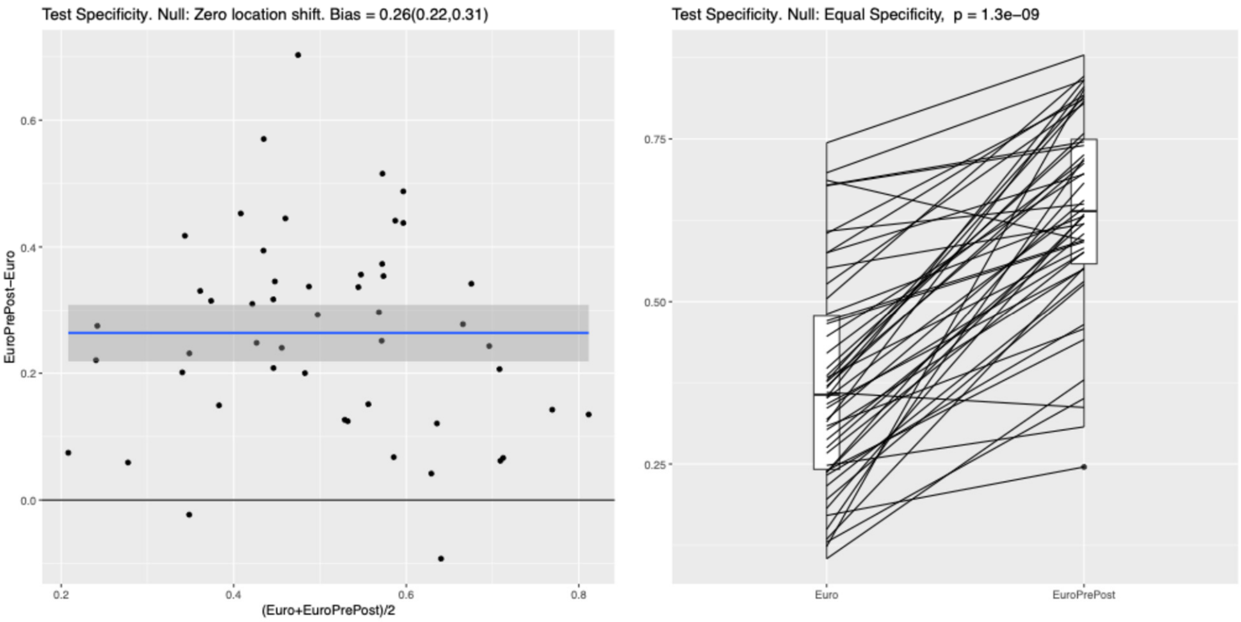

Figure S4. Neural Network: Comparison of test specificity at 85% sensitivity between Model II (EuroScore variables + additional pre- and postoperative variables) and Baseline Model (EuroScore variables alone). Legend: This figure compares test specificity at 85% sensitivity for a neural network classifier using the baseline EuroSCORE II variables ("Euro") versus an extended feature set including both preoperative and postoperative variables ("EuroPrePost," Model II). The left panel shows specificity differences across 100 random train-test splits, with a mean improvement of 0.26 (95% CI: 0.22–0.31, blue line). The right panel presents paired boxplots of specificity values, with connecting lines indicating results from the same split. The difference is statistically significant ($p = 1.3 \times 10^{-9}$, Wilcoxon signed-rank test), highlighting the strong impact of postoperative data on predictive performance and survivor classification accuracy.

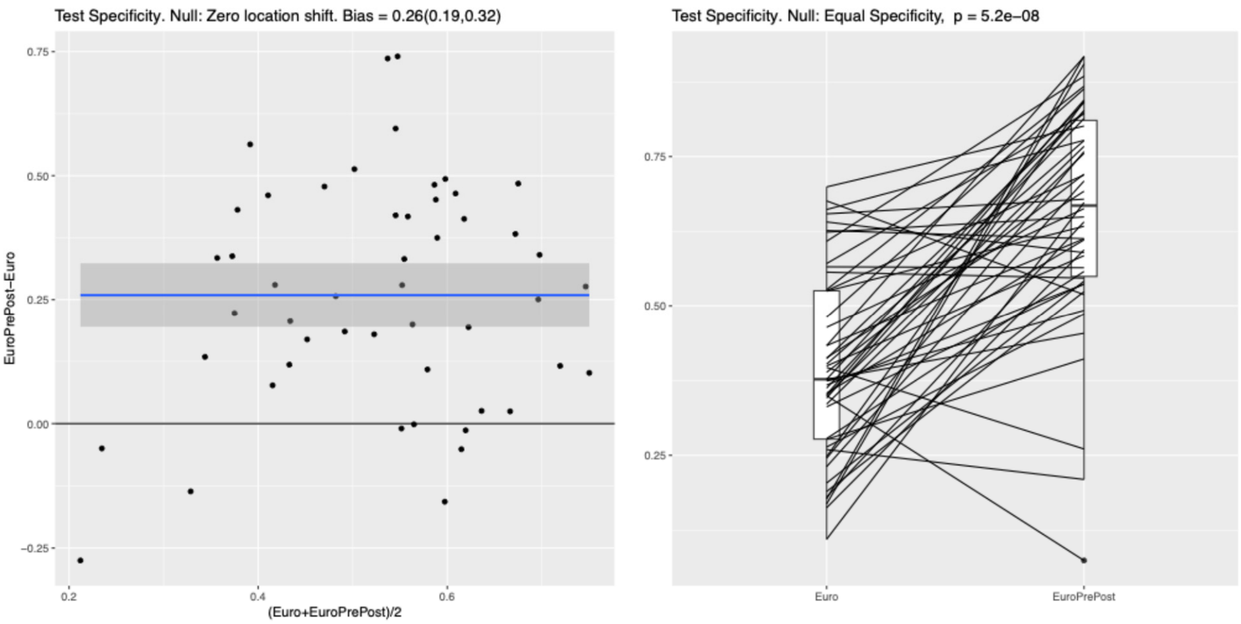

Figure S5. Logistic Regression: Comparison of test specificity at 85% sensitivity between Model II (EuroScore variables + additional pre- and postoperative variables) and Baseline Model (EuroScore variables alone). Legend: This figure presents the comparison of test specificity at 85% sensitivity for logistic regression models trained on the baseline EuroSCORE II variables ("Euro") and an extended feature set including both preoperative and postoperative variables ("EuroPrePost," Model II). The left panel shows the distribution of specificity differences across 100 random train-test splits, with a mean improvement of 0.26 (95% CI: 0.19–0.32, blue line). The right panel displays paired specificity values as boxplots, where each line connects results from the same split. The improvement is statistically significant ($p = 5.2 \times 10^{-8}$, Wilcoxon signed-rank test), indicating that even a linear model like logistic regression can benefit substantially from the inclusion of postoperative data in short-term mortality prediction.
